# Combination of ELISA screening and seroneutralisation tests to expedite Zika virus seroprevalence studies

**DOI:** 10.1186/s12985-018-1105-5

**Published:** 2018-12-27

**Authors:** Elif Nurtop, Paola Mariela Saba Villarroel, Boris Pastorino, Laetitia Ninove, Jan-Felix Drexler, Yelin Roca, Bouba Gake, Audrey Dubot-Peres, Gilda Grard, Christophe Peyrefitte, Stéphane Priet, Xavier de Lamballerie, Pierre Gallian

**Affiliations:** 1 Unité des Virus Émergents (UVE: Aix-Marseille Univ – IRD 190 – Inserm 1207 – IHU Méditerranée Infection), Marseille, France; 2 Virología II, Centro Nacional de Enfermedades Tropicales (CENETROP), Santa Cruz de la Sierra, Bolivia; 3 Institute of Virology, Charité-Universitätsmedizin Berlin, Berlin, Germany; 4 Centre Pasteur du Cameroun, Yaoundé, Cameroon; 5 National Reference Centre for Arboviruses, French Armed Forces Biomedical Research Institute, Marseille, France; 6 Laboratoire de Virologie, Établissement Français du Sang Alpes Méditerranée (EFS), Marseille, France

**Keywords:** Zika virus, Seroepidemiology, Virus neutralization test

## Abstract

Here we propose a strategy allowing implementing efficient and practicable large-scale seroepidemiological studies for Zika Virus (ZIKV). It combines screening by a commercial NS1 protein-based Zika IgG ELISA, and confirmation by a cytopathic effect-based virus neutralization test (CPE-based VNT). In post-epidemic samples from Martinique Island blood donors (a population with a dengue seroprevalence above 90%), this strategy allowed reaching specificity and sensitivity values over 98%. The CPE-based VNT consists of recording CPE directly under the optical microscope, which is easy to identify with ZIKV strain H/PF/2013 at day 5 pi. Overall, considered that CPE-based VNT is cost effective and widely automatable, the NS1 protein-based Zika IgG ELISA+CPE-based VNT combination strategy represents a convenient tool to expedite ZIKV seroprevalence studies.

**Electronic supplementary material:**

The online version of this article (10.1186/s12985-018-1105-5) contains supplementary material, which is available to authorized users.

## Introduction

Zika virus (ZIKV) is an enveloped RNA virus (family *Flaviviridae,* genus *Flavivirus*) [1] of African origin, first identified as the aetiological agent of mild arboviral fever [2] and mainly transmitted to human by the bite of infected *Aedes* mosquitoes [3]. Beginning in Yap Island (in 2007), ZIKV has been responsible for large outbreaks in French Polynesia and other Pacific Islands (2013–2015), then in South America and the Caribbean’s (from 2014 to 2015) [4]. Recently, intra-uterine [5] and sexual routes of transmission have been identified [6] and severe neurological presentations have been reported in adults (myelitis, encephalitis, Guillain-Barré syndrome) [7] and in foetus (including microcephaly) [8].

Due to frequent asymptomatic infections (ranging between 29 to 82% in different populations) [9], the survey of ZIKV clinically suspected cases is poorly adapted to estimate the attack rate during ZIKV outbreaks. This information can be provided by seroprevalence studies [10]. However, antigenic cross-reactivity between ZIKV and other flaviviruses (particularly dengue virus) makes seroprevalence studies challenging and requires wide use of seroneutralization assays [11].

We implemented a strategy aiming at facilitating ZIKV seroprevalence studies. It relies on primary testing using an ELISA assay to allow convenient screening of large series of samples, followed by a virus neutralization test (VNT) for confirmation of equivocal and positive ELISA results. For VNT, we chose a format usable for large series, combining a 96-well culture format and a simple cytopathic effect (CPE) based readout.

Here, we describe the performances of our testing strategy [ELISA screening+VNT confirmation]. We first evaluated our VNT versus classical Plaque Reduction Neutralization Test (PRNT) and then studied the sensitivity and specificity of the [ELISA screening+VNT confirmation] strategy in blood donors from Martinique Island (i.e. in a population heavily exposed to dengue infection) [12], before the arrival of ZIKV, and after the ZIKV outbreak.

## Methods

### Serum samples

In order to compare VNT and PRNT, we used a panel of sera from blood donors of different origins: 90 from Martinique Island and 10 from Guadeloupe Island (all collected after the 2015–2016 outbreak due to ZIKV Asian genotype); 7 from Cameroon (where low ZIKV seropositivity [13] is presumably linked with the circulation of ZIKV African genotype); 35 from Metropolitan France (where no ZIKV or DENV circulation has been reported). For the performance testing of ELISA+VNT combination strategy, a total of 592 serum specimens collected from Martinique Island (collected before and after the 2015–2016 ZIKV outbreak) were included. A total of 13 serum specimens collected from laboratory staff at least one year after vaccination against several flaviviruses, including Yellow Fever, Japanese Encephalitis and Tick-Borne Encephalitis virus, were included to test the cross reactivity of CPE-based VNT. The consent of the each participant was taken prior to any laboratory testing.

### NS1 protein-based Zika IgG EUROIMMUN ELISA

NS1 protein-based Zika IgG ELISA was performed for all samples according to the manufacturer’s instructions. Semiquantitative ratios were calculated and specimens with a ratio value < 0.8 were considered as IgG negative; those with a ratio ≥ 1.1 were considered as positive and those with a ratio ≥ 0.8 to < 1.1 were recorded as equivocal according to the Euroimmun recommendations.

### Virus neutralisation test (VNT)

VNT was carried out with H/PF/2013 strain of the ZIKV in 96-well plates containing confluent Vero cells (ATCC CCL-81). H/PF/2013 strain of the ZIKV was selected as this strain gives clear CPE on Vero cells. We used 6 dilutions of each heat inactivated serum (1:10 to 1:320 –corresponding to final testing dilutions 1:20 to 1:640, see below), allowing testing 16 samples or controls per plate. All the serum dilutions and virus additions were done with the automated system (Eppendorf EpMotion 5075). In each VNT, a strong, assured positive control serum from French National Reference Centre for Arboviruses was used (PRNT90 titre: 2560). Sera were mixed vol/vol with 100 TCID50/reaction of ZIKV and incubated 1 h at 37 °C. Then the serum+virus mixture was transferred onto the confluent cell monolayer in duplicate. Two possible readout strategies were evaluated: CPE-based VNT and PCR-based VNT. CPE-based VNT relied on CPE observation directly with light microscopy at day 5 pi. After the 5 days incubation period, gross cytopathic effect can be observed on Vero cells with H/PF/2013 strain of ZIKV. As seen in the Additional File 1, after the incubation period, distinguishing the presence/absence of viral cytophatic effect caused by H/PF/2013 strain of ZIKV is straightforward and easily readable under light microscope. Dilutions of serum associated with CPE were considered as negative, while the absence of CPE indicated a complete neutralisation of ZIKV inoculum (positive result). Consequently, Virus Neutralisation Titre referred to VNT100 and is described as the highest dilution of serum that neutralized virus growth. PCR-based VNT relied on RNA extraction from culture supernatants at day 3 pi and subsequent ZIKV qRT-PCR detection. For the qRT-PCR, ThermoFisher Scientific Express One-Step Superscript qRT-PCR kit with primers 5’-GRGCYCGGCCAATCAG-3′ & 5’-AARGACGGGAGRTCCATTGTG-3′ and the probe FAM-CGCCACCAAGATGA-MGB were used [14]. After the qRT-PCR, the viral load in each serum dilution and the positive control were assessed. As for PCR-based VNT, the presence/absence of viral replication was recorded. All sample dilutions associated with a viral load superior to that of our positive control sample were considered as negative while a viral load inferior or equal to that of our positive control sample was the hallmark of a positive dilution. Consequently, both PCR- and CPE-based VNT were based on the presence or complete absence of virus growth (VNT100). For both tests, serum specimens with a titre ≥40 were considered positive according to the recommendations of the French National Reference Centre for Arboviruses [10].

### Plaque reduction neutralization test (PRNT)

PRNT was adapted from previously described protocols [15, 16]. It was carried out with H/PF/2013 strain (60 PFU/reaction corresponding to ~ 86 TCID/reaction) in 6 well plates containing confluent Vero cell monolayer and 6 dilutions of each serum (1:5 to 1:160 –corresponding to final testing dilutions 1:10 to 1:320, see below). After one-hour incubation at 37 °C, the vol/vol serum+virus mixture was transferred onto confluent Vero cells monolayer in 6 well plates. Following incubation, inoculums were removed and cell monolayers were immediately covered with carboxymethylcellulose (CMC)/DMEM overlay. Plaques were counted at day 5 pi. Serum specimens with 90% (PRNT90) or 50% (PRNT50) reduction of the number of plaques at titres ≥10 were recorded as positive according to the recommendation of Centers for Disease Control and Prevention [17, 18]. (Additional file 2 shows image of plaque of PRNT at day 5 pi).

## Results & discussion

### Validation of the virus neutralisation test (VNT)

In order to estimate the analytical specificity and sensitivity of the VNT for ZIKV, VNT results of the panel were compared with the plaque reduction neutralization test (PRNT) which is considered to be the “gold standard technique”.

#### Comparison of VNT and PRNT results

**Fig. 1:**
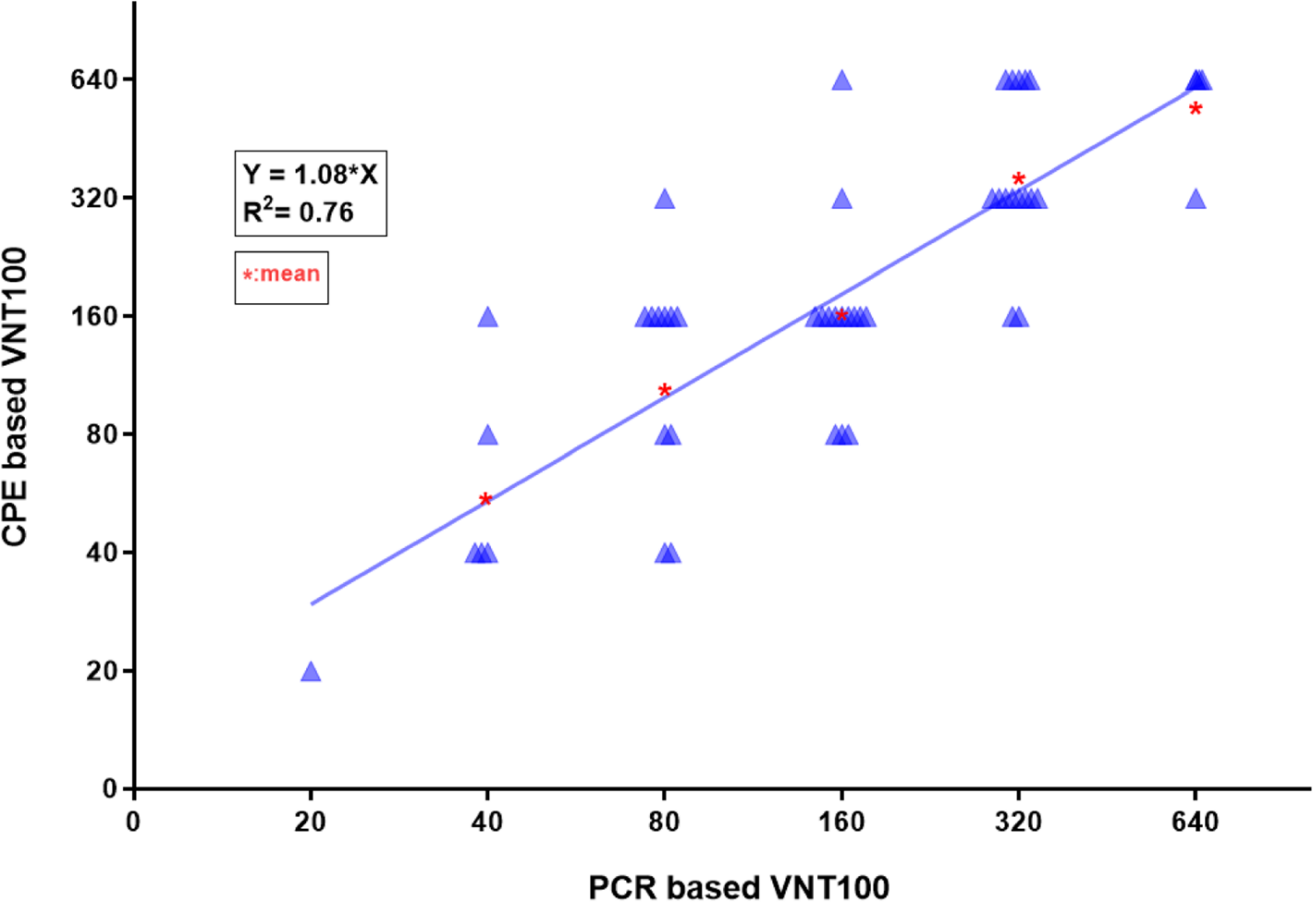
Virus Neutralization Titre (VNT100) comparison of PCR-based and Cytopathic Effect (CPE)-based Virus Neutralization Test (VNT). VNT100 were described in log10. Titres in the axes were described as antilogs. Mean values of CPE-based VNT titres corresponding to each PCR-based VNT titre are shown as red stars. Equation of the linear regression curve and R^2^ value indicate that titres from the two methods used are correlated

The same results (positive/negative) were obtained using CPE- and PCR-based VNTs. Titres were also highly correlated (Fig. 1). Accordingly, for the sake of convenience and cost effectiveness, CPE-based VNT was used in the subsequent steps of our study. For PCR and CPE-based VNT, the quality of the assay was determined by calculating the Zˈ factor. For both inter-well and inter-experiment, Zˈ factor was 1 which indicated that the variation between duplicates and experiments is minimum.

For VNT, we tested threshold titres at 20, 40, and 80. As expected, a threshold at 20 resulted in a decrease of the specificity and a threshold at 80 resulted in a decrease of sensitivity but also in 100% specificity (Additional files 3 and 4). Altogether, the threshold at 40 represented a good compromise. For PRNT, titres ≥10 were recorded as positive according to the recommendation of Centers for Disease Control and Prevention [17, 18]. Additionally, the comparison of the results obtained using titres ≥10 or ≥ 20 for both PRNT50 and PRNT90, confirmed that PRNT90 with a titre ≥10 was associated with a higher sensitivity and specificity in VNT (98.1% sensitivity and 98.8% specificity) (Additional file 5).

When CPE-based VNT was compared with PRNT, the best correlation was obtained with PRNT90 (R^2^ was 0.83 with PRNT90 and 0.78 with PRNT50) (Additional files 6 and 7). Sensitivity and specificity of CPE-based VNT were 98.1 and 98.8% respectively, with reference to PRNT90 (Table 1).

**Table 1:** Comparison of VNT and PRNT assays for a panel of 142 samples

	PRNT50		PRNT90	
Comparison of VNT and PRNT	Positive (titre≥10)	Negative (titre< 10)	Positive (titre≥10)	Negative (titre< 10)
Comparison of VNT and PRNT	Positive (titre≥40)	1	51	1
Comparison of VNT and PRNT	Negative (titre<40)	81	1	89
Sensitivity of VNT (95% CI)	85% (51/60) (72.9–92.4%)		98.1% (51/52) (88.4–99.9%)	
Specificity of VNT (95% CI)	98.7% (81/82) (92.4–99.9%)		98.8% (89/90) (93.1–99.9%)	

Cytopathic Effect (CPE) based Virus Neutralization Test (VNT) was compared with Plaque Reduction Neutralization Test with either 50% or 90% End-Point Reduction (PRNT50 or PRNT90, respectively). Sensitivity and specificity of the CPE-based VNT were calculated with reference to PRNT50 or PRNT90 used as a gold standard.

When the sensitivity and specificity of CPE-based VNT was compared in Dengue ELISA negative and Dengue ELISA positive group, similar sensitivity and specificity was observed (100 and 98% of sensitivity, 100 and 97.7% of specificity, respectively) (Additional file 8). Also it was found that the sensitivity and specificity of CPE-based VNT is higher in sera with strong ZIKV seropositivity (97.1% sensitivity and 100% specificity in sera with a titre ≥160 and 94.4% sensitivity and 98.9% specificity in sera with titre 40–80) (Additional file 9).

#### VNT in individuals with vaccine flaviviral immunisation

To better evaluate the extent of cross-reactivity of VNT in case of previous immunisation against various flaviviruses, we tested a total of 13 serum specimens collected from laboratory staff immunized against different flaviviruses at least 1 year ago prior to serum collection. Three had received Yellow fever vaccine only; five Yellow fever and Tick Born Encephalitis vaccines; one Yellow fever and Japanese Encephalitis vaccines; four Yellow fever, Tick Born Encephalitis and Japanese Encephalitis vaccines. None of the samples provided ZIKV CPE-based VNT positive result, highlighting the robustness of the test specificity.

### Performance of the [ELISA screening + VNT confirmation] strategy versus systematic VNT

The performance of the combined [ELISA screening+VNT confirmation] strategy was evaluated in blood donors from Martinique Island. This choice was justified by the fact that *(i)* we knew that this population had been heavily exposed to the circulation of ZIKV in 2015–2016 [10], *(ii)* we also knew that seroprevalence of dengue is ~ 90% in this population [12], *(iii)* we had samples collected before the arrival of ZIKV (9–10 September 2013) and 6 months after the end of the outbreak (25 January-14 February 2017). Accordingly, all samples were tested using both the ELISA (NS1 protein-based Zika IgG EUROIMMUN assay, Lübeck, Germany) and the CPE-based VNT, and results of the [ELISA screening+VNT confirmation] strategy were compared with those obtained using VNT as a first line test.

#### Specificity study (pre-epidemic cohort)

The pre-epidemic study was conducted with 144 sera collected before the ZIKV outbreak in Martinique Island: 142 were VNT negative and 2 were VNT positive (titres 40 and 80; samples also positive with PRNT90 and PRNT50). These positive results might be explained either by cross reactivity with another flavivirus (most probably dengue), or by an actual ZIKV infection acquired outside Martinique Island. Whatever the explanation, this result indicates that the absolute specificity of both VNT and PRNT tests is at least 98.6%.

The NS1 protein-based Zika IgG ELISA test was positive (ratio ≥ 1.1) for 55 specimens (38.2%) of which 2 (1.4%) were confirmed by VNT (and correspond to those described in the previous paragraph). It was equivocal (0.8 ≤ ratio < 1.1) for 8 samples and negative in 81 samples (all negative in VNT) (Table 2). Accordingly, the specificity of the [ELISA screening+VNT confirmation] strategy was identical to that of the VNT test alone (≥98.6%). The specificity of the NS1 protein-based Zika IgG ELISA alone was 62.7% (89/142).

**Table 2:** ELISA and VNT results in pre- and post-epidemic Martinique samples

	ELISA positive		ELISA equivocal		ELISA negative	
	VNT positive	VNT negative	VNT positive	VNT negative	VNT positive	VNT negative
Pre-epidemic study (*n* = 144)	2	53	0	8	0	81
Post-epidemic study (*n* = 448)	229	49	8	16	5	141

Blood donations were tested using the NS1 protein-based Zika IgG EUROIMMUN ELISA, (Lübeck, Germany) and the CPE-based VNT.

#### Sensitivity study (post-epidemic cohort)

448 blood specimens collected after the ZIKV outbreak were tested with the same strategy as described above: 206 were VNT negative and 242 were VNT positive.

The anti-ZIKV IgG ELISA test was positive for 237 specimens (62.05%) and 229 of them (51.1% of the total samples) were confirmed by VNT. It was equivocal for 24 samples, of which 8 (1.7%) were positive by CPE-based VNT, and negative for 146 samples of which 5 (1.12%) were positive by CPE-based VNT (Table 2). Accordingly, in the post-epidemic cohort, the strategy combining [ELISA screening+VNT confirmation] had a sensitivity of 98% (237 in 242 positives detected using VNT as a first line assay).

## Conclusion

In this study we proposed a strategy for Zika seroepidemiological studies, which comprises a first line screening with anti-ZIKV IgG ELISA followed by a confirmation of non-negative samples by CPE-based VNT.

We confirmed the limited specificity of the NS1 protein-based Zika IgG EUROIMMUN ELISA (62.7%) in sera collected before the ZIKV outbreak in Martinique Island where the Dengue is prevalent, presumably due to the cross-reactive ZIKV and DENV antibodies [19, 20]. Our findings are in accordance with a recent report which detected low specificity (45%) of NS1 protein-based Zika IgG ELISA in sera of blood donor collected before the ZIKV outbreak in Brazil [21]. This result points out the importance of confirmation of the NS1 protein-based Zika IgG EUROIMMUN ELISA with neutralisation assays especially in dengue endemic areas.

This [ELISA screening+VNT confirmation] strategy was associated with sensitivity and specificity values around 98%, when tested in a population of blood donors of Martinique Island in which dengue seroprevalence is over 90%. Until now, various neutralisation assays for ZIKV with different end-point reading strategies have been proposed as an alternative to PRNT, such as the usage of viability test with the 3-[4,5-dimethyl-2-thiazolyl]-2,5-diphenyl-2H-tetrazolium bromide-(MTT) [22], image based fluorescent neutralisation tests [23], end-point assessment with real time PCR [24], or usage of luciferase ZIKV in neutralisation [25]. However, these tests are dependent on the usage of RNA extraction and qRT-PCR, or fluorescence or luminescence plate reader, which increase the global cost of the assays. Nevertheless, CPE-based VNT only requires the usage of a ZIKV strain that gives clear CPE.

In here, we described a cost effective methodology for seroepidemiological studies. This methodology was not designed or intended for individual diagnosis. It represents a convenient, rapid and cost-effective method to process large series with the aim of producing a reliable picture of the epidemiological situation as previously demonstrated in Martinique Island, Cameroon and Bolivia [10, 13, 26].

## Additional files

Additional file 1: Light microscopy image of a VNT assay at day 5 pi. (DOCX 3995 kb)

Additional file 2: Plaque Reduction Neutralisation Test of ZIKV at day 5 pi. (DOCX 9182 kb)

Additional file 3: Comparison of VNT (threshold set at 20) and PRNT assays for a panel of 142 samples. (DOCX 12 kb)

Additional file 4: Comparison of VNT (threshold set at 80) and PRNT assays for a panel of 142 samples. (DOCX 14 kb)

Additional file 5: Sensitivity and specificity comparison of VNT and PRNT (threshold was set as 10 and 20) for a panel of 142 samples. (DOCX 15 kb)

Additional file 6: Comparison of CPE-based Virus Neutralization Titre100 and PRNT50 titres. (DOCX 172 kb)

Additional file 7: Comparison of CPE-based Virus Neutralization Titre100 and PRNT90 titres. (DOCX 168 kb)

Additional file 8: Specificity and sensitivity of VNT in DENGUE ELISA negative and positive samples. (DOCX 14 kb)

Additional file 9: Specificity and sensitivity of VNT in low ZIKV seropositivity (titre 40–80) and strong ZIKV positivity (titre ≥160) compared to the PRNT90. (DOCX 14 kb)

## Abbreviations

CPE: Cytopathic effect
ELISA: The enzyme-linked immunosorbent assay
Pi: Post-infection
PFU: Plaque Forming Unit
PRNT: Plaque Reduction Neutralization Test
TCID: Tissue Culture Infective Dose
VNT: Virus Neutralization Test
ZIKV: Zika Virus
