# Correction to: Combination of ELISA screening and seroneutralisation tests to expedite Zika virus seroprevalence studies

**DOI:** 10.1186/s12985-019-1118-8

**Published:** 2019-01-18

**Authors:** Elif Nurtop, Paola Mariela Saba Villarroel, Boris Pastorino, Laetitia Ninove, Jan Felix Drexler, Yelin Roca, Bouba Gake, Audrey Dubot-Peres, Gilda Grard, Christophe Peyrefitte, Stéphane Priet, Xavier de Lamballerie, Pierre Gallian

**Affiliations:** 1 Unité des Virus Émergents (UVE: Aix-Marseille Univ – IRD 190 – Inserm 1207– IHU Méditerranée Infection), Marseille, France; 2 Virología II, Centro Nacional de Enfermedades Tropicales (CENETROP), Santa Cruz de la Sierra, Bolivia; 3 Institute of Virology, Charité-Universitätsmedizin Berlin, Berlin, Germany; 4 Centre Pasteur du Cameroun, Yaoundé, Cameroon; 5 National Reference Centre for Arboviruses, French Armed Forces Biomedical Research Institute, Marseille, France; 6 Laboratoire de Virologie, Établissement Français du Sang Alpes Méditerranée (EFS), Marseille, France

**Correction to: Virol J**

**https://doi.org/10.1186/s12985-018-1105-5**

In the original publication of this article [1], Table 1 has some errors. The correct one is below:

**Table 1:** Comparison of VNT and PRNT assays for a panel of 142 samples

VNT	PRNT50		PRNT90	
	Positive (titre ≥10)	Negative (titre < 10)	Positive (titre ≥10)	Negative (titre < 10)
Positive (titre ≥40)	51	1	51	1
Negative (titre < 40)	9	81	1	89
Sensitivity of VNT (95% CI)	85% (51/60) (72.9–92.4%)		98.8% (51/52) (88.4–99.9%)	
Specificity of VNT (95% CI)	98.7% (81/82) (92.4–99.9%)		98.8% (89/90) (93.1–99.9%)	

The publisher apologizes to the readers and authors for the inconvenience.

The original publication has been corrected.
