# Supplementary material for: Combination of ELISA screening and seroneutralisation tests to expedite Zika virus seroprevalence studies
Source: Virol J. 2018 Dec 27;15:192. doi: 10.1186/s12985-018-1105-5 (PMC6307276; doi:10.1186/s12985-018-1105-5)
Supplement: Supplementary file 1 — Light microscopy image of a VNT assay at day 5 pi. (DOCX 3995 kb) [file 12985_2018_1105_MOESM1_ESM.docx]

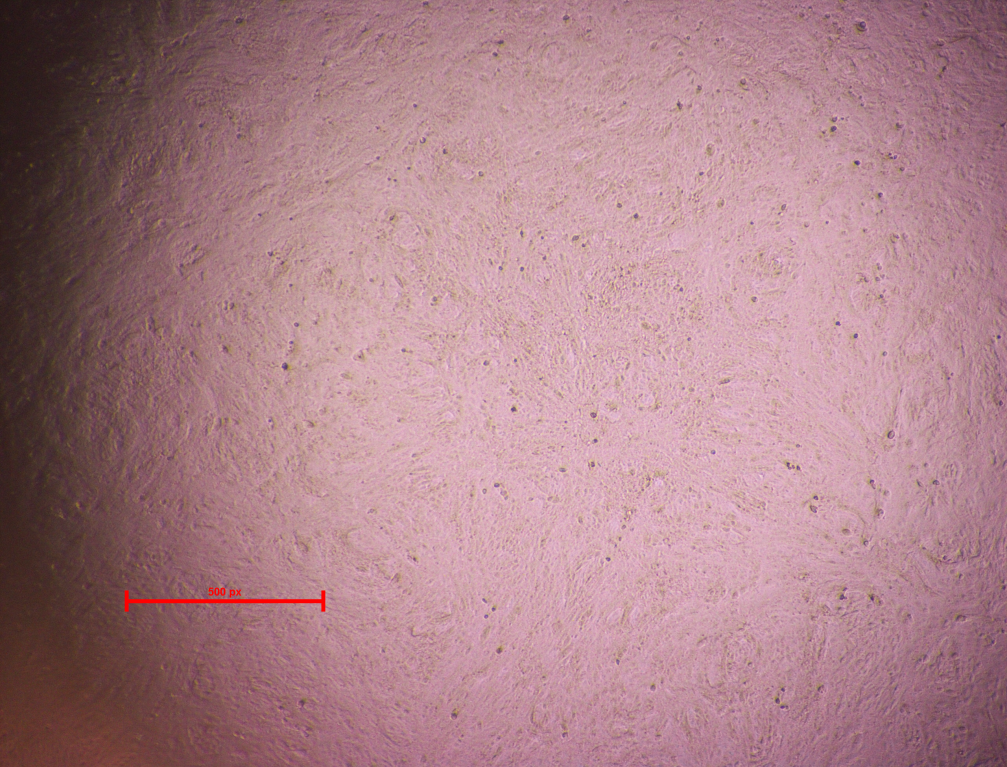

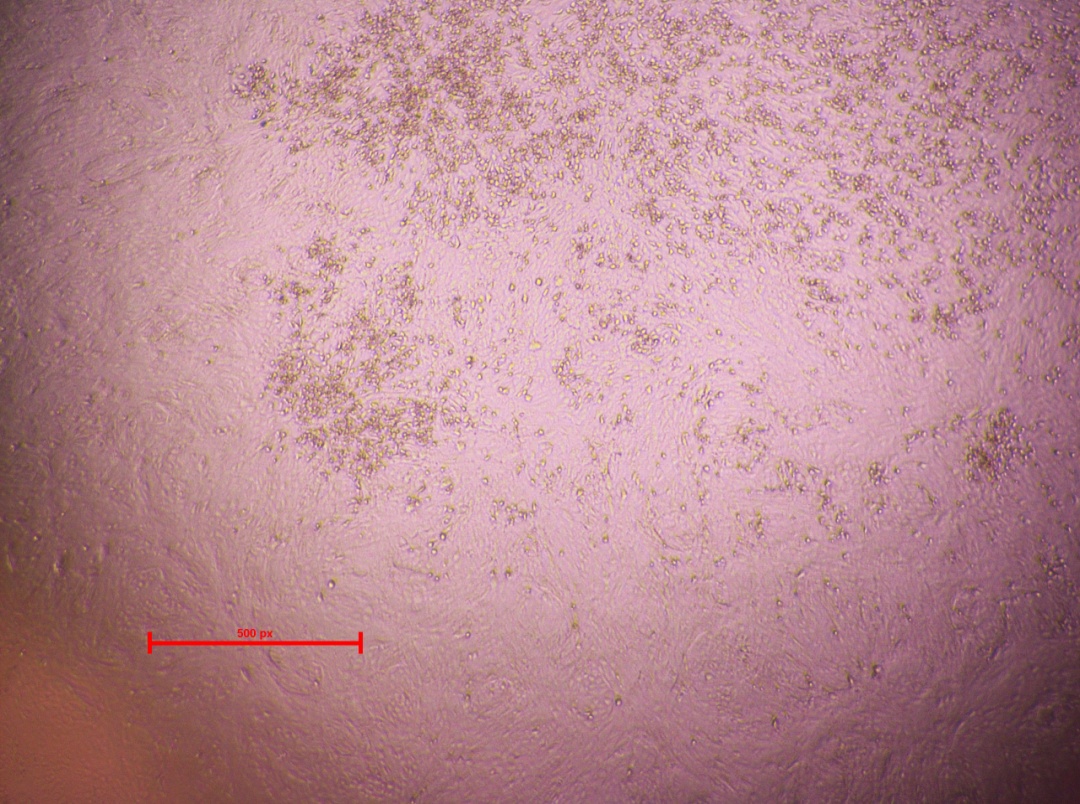

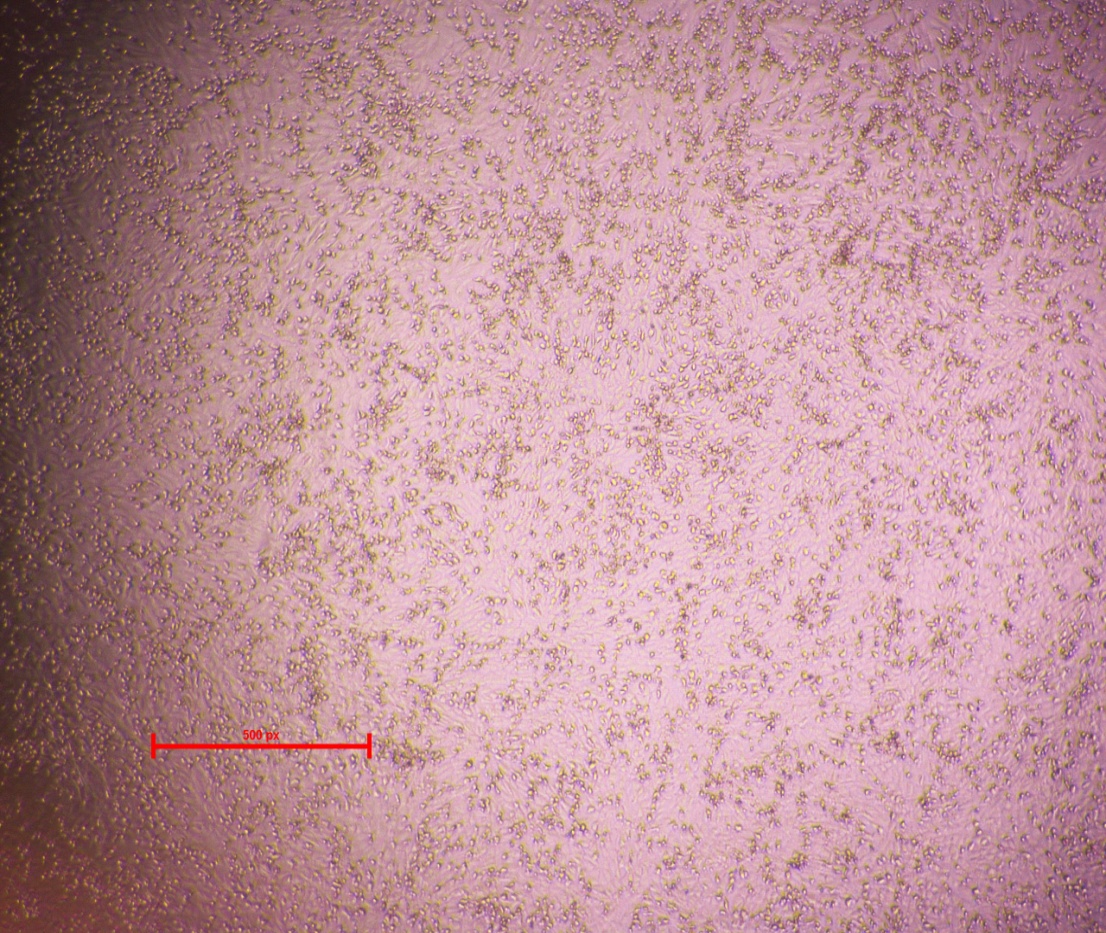

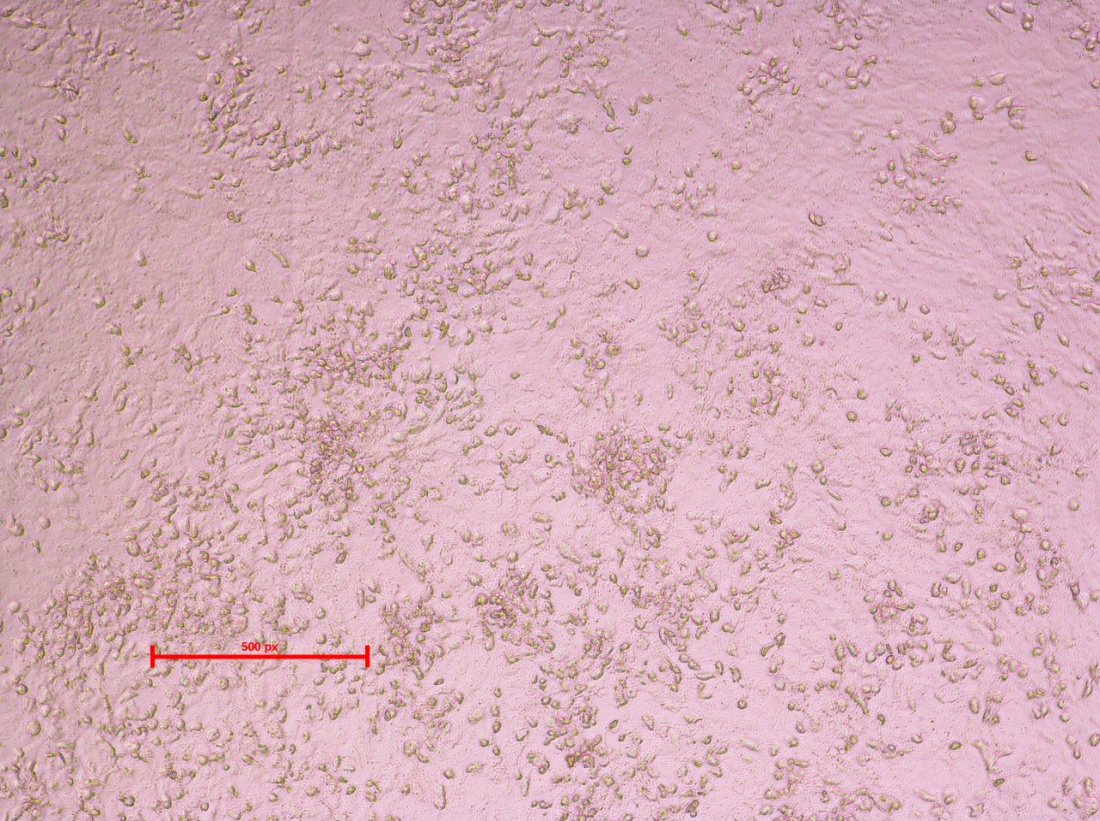


**D**

**C**

**B**

**A**

**Additional file 1.** Light microscopy image of a VNT assay at day 5 pi. (A) Light microscopy image of a well with no CPE. (B,C,D) The images of well with CPE
