# Supplementary material for: Combination of ELISA screening and seroneutralisation tests to expedite Zika virus seroprevalence studies
Source: Virol J. 2018 Dec 27;15:192. doi: 10.1186/s12985-018-1105-5 (PMC6307276; doi:10.1186/s12985-018-1105-5)
Supplement: Supplementary file 2 — Plaque Reduction Neutralisation Test of ZIKV at day 5 pi. (DOCX 9182 kb) [file 12985_2018_1105_MOESM2_ESM.docx]

**A**

**B**

**
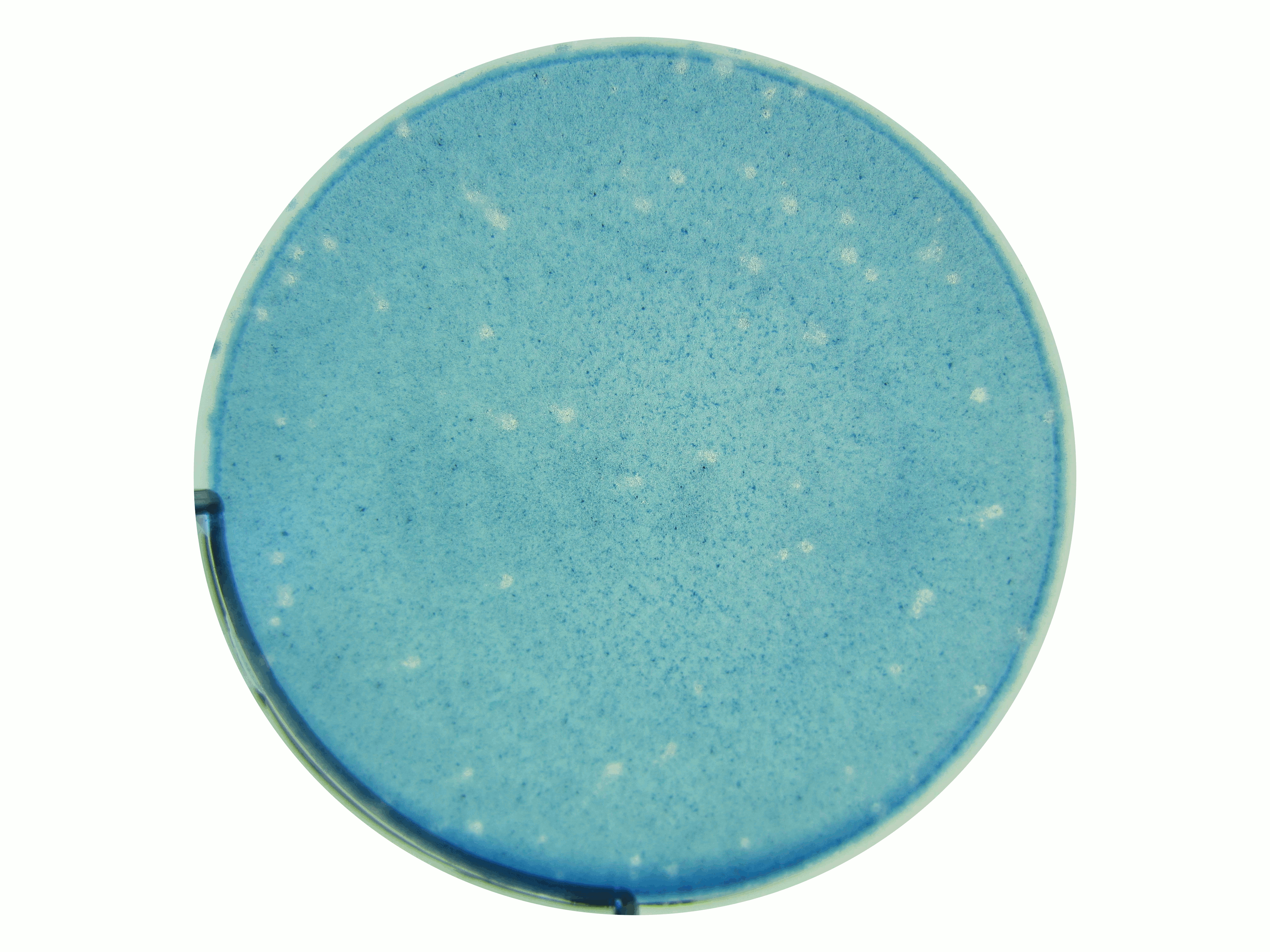

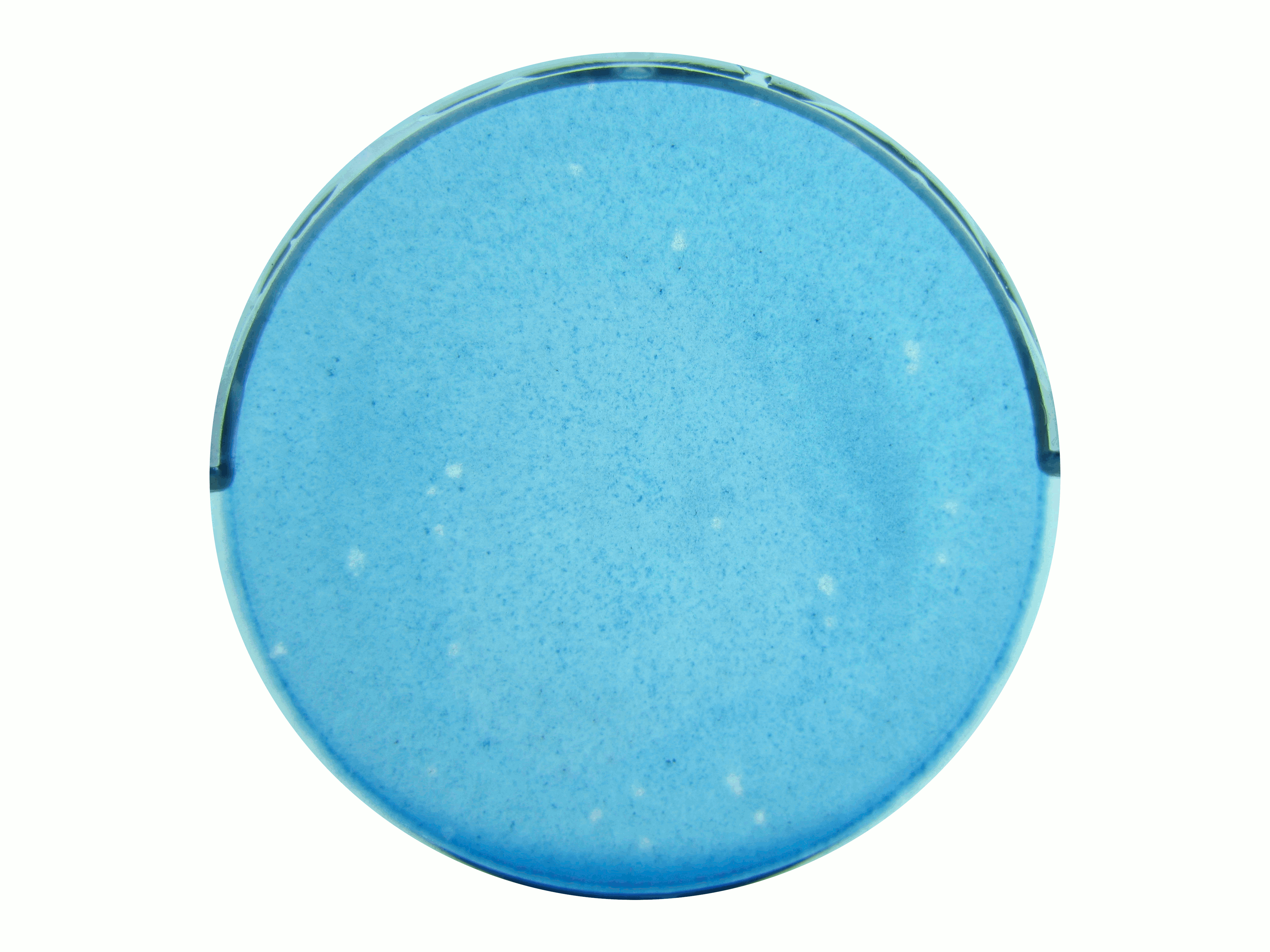

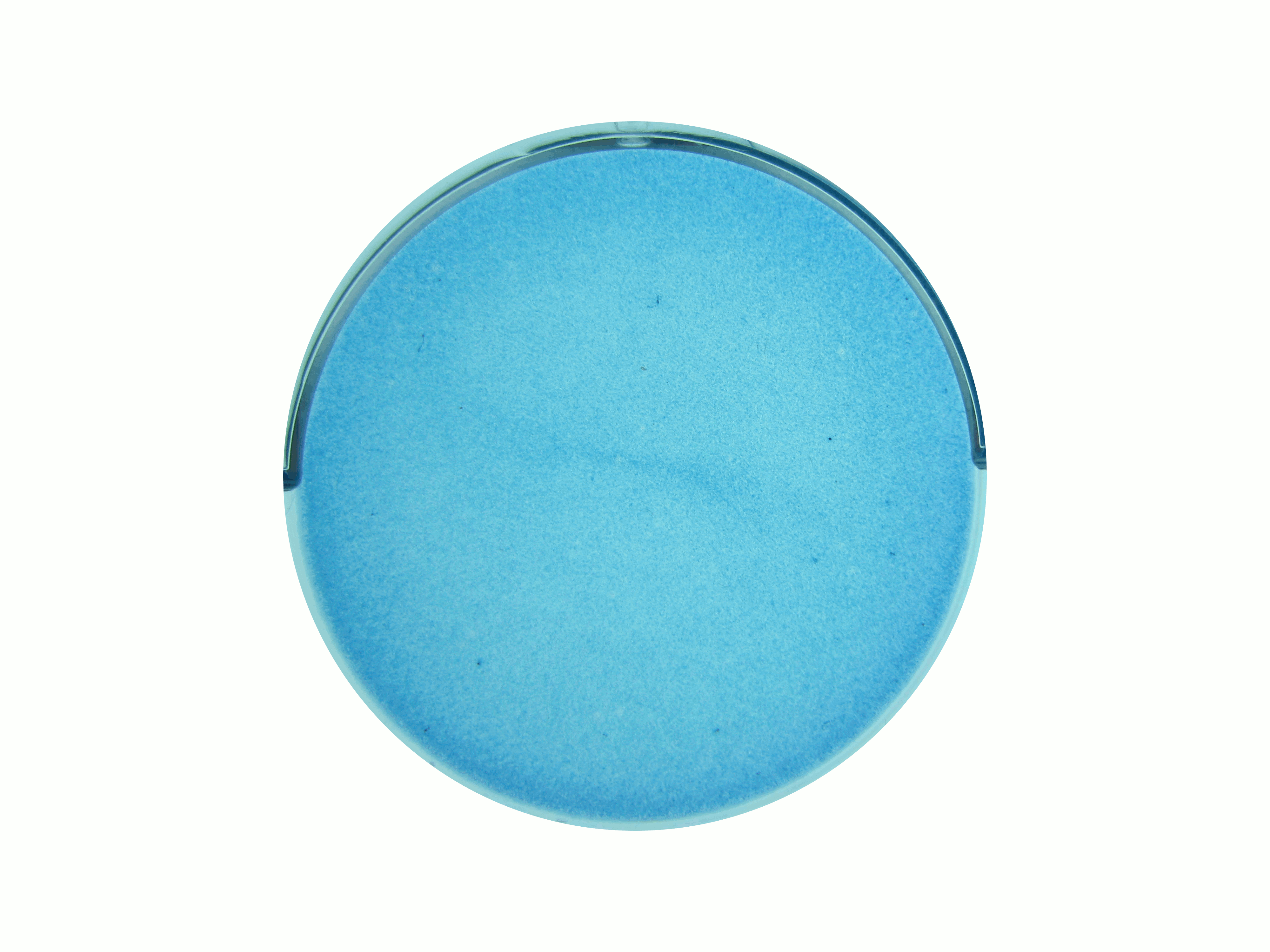
**

**C**

**Additional file 2.** Plaque Reduction Neutralisation Test of ZIKV at day 5 pi. (A) A well of a 6 well plate of negative control showing 58 plaques. (B,C) A well of 6 well plate neutralized by ZIKV positive serum showing 20 plaques and no plaque, respectively
