# Supplementary material for: Combination of ELISA screening and seroneutralisation tests to expedite Zika virus seroprevalence studies
Source: Virol J. 2018 Dec 27;15:192. doi: 10.1186/s12985-018-1105-5 (PMC6307276; doi:10.1186/s12985-018-1105-5)
Supplement: Supplementary file 3 — Comparison of VNT (threshold set at 20) and PRNT assays for a panel of 142 samples. (DOCX 12 kb) [file 12985_2018_1105_MOESM3_ESM.docx]

**Additional file 3.** Comparison of VNT (threshold set at 20) and PRNT for a panel of 142 samples.

|  | **PRNT50** | | **PRNT90** | |
| --- | --- | --- | --- | --- |
| **VNT100** | Positive (titre≥10) | Negative (titre<10) | Positive (titre≥10) | Negative (titre<10) |
| Positive (titre≥20) | 53 | 1 | 51 | 3 |
| Negative (titre<20) | 7 | 81 | 1 | 87 |
| Sensitivity of VNT (95% CI) | 88.3 % (53/60)  (76.82%-94.78%) | | 98.1 % (51/52)  (88.4%-99.9%) | |
| Specificity of VNT (95% CI) | 98.7% (81/82)  (92.5%-99.9%) | | 96.6% (87/90)  (89.9%-99.1%) | |

Cytopathic Effect (CPE) based Virus Neutralization Test (VNT) was compared with Plaque Reduction Neutralization Test with either 50% or 90% End-Point Reduction (PRNT50 or PRNT90, respectively). Sensitivity and specificity of the CPE-based VNT were calculated with reference to PRNT50 or PRNT90 used as a gold standard.
