# Supplementary material for: Combination of ELISA screening and seroneutralisation tests to expedite Zika virus seroprevalence studies
Source: Virol J. 2018 Dec 27;15:192. doi: 10.1186/s12985-018-1105-5 (PMC6307276; doi:10.1186/s12985-018-1105-5)
Supplement: Supplementary file 4 — Comparison of VNT (threshold set at 80) and PRNT assays for a panel of 142 samples. (DOCX 14 kb) [file 12985_2018_1105_MOESM4_ESM.docx]

**Additional file 4.** Comparison of VNT (threshold set at 80) and PRNT for a panel of 142 samples

|  | **PRNT50** | | **PRNT90** | |
| --- | --- | --- | --- | --- |
| **VNT100** | Positive (titre≥10) | Negative (titre<10) | Positive (titre≥10) | Negative (titre<10) |
| Positive (titre≥80) | 47 | 0 | 47 | 0 |
| Negative (titre<80) | 13 | 82 | 5 | 90 |
| Sensitivity of VNT (95% CI) | 78.3% (47/60)  (65.4%-87.5%) | | 90.4 % (47/52)  (78.2%-96.4%) | |
| Specificity of VNT (95% CI) | 100% (82/82)  (94.4%-100%) | | 100% (90/90)  (94.9%-100%) | |

Cytopathic Effect (CPE) based Virus Neutralization Test (VNT) (threshold ≥80) was compared with Plaque Reduction Neutralization Test with either 50% or 90% End-Point Reduction (PRNT50 or PRNT90, respectively). Sensitivity and specificity of the CPE-based VNT were calculated with reference to PRNT50 or PRNT90 used as a gold standard.
