# Supplementary material for: Combination of ELISA screening and seroneutralisation tests to expedite Zika virus seroprevalence studies
Source: Virol J. 2018 Dec 27;15:192. doi: 10.1186/s12985-018-1105-5 (PMC6307276; doi:10.1186/s12985-018-1105-5)
Supplement: Supplementary file 5 — Sensitivity and specificity comparison of VNT and PRNT (threshold was set as 10 and 20) for a panel of 142 samples. (DOCX 15 kb) [file 12985_2018_1105_MOESM5_ESM.docx]

**Additional file 5.** Sensitivity and specificity comparison of VNT and PRNT (threshold was set as 10 and 20) for a panel of 142 samples

|  | **PRNT (titre≥10)** | | | | **PRNT (titre ≥20)** | | | |
| --- | --- | --- | --- | --- | --- | --- | --- | --- |
|  | **PRNT50** | | **PRNT90** | | **PRNT50** | | **PRNT90** | |
| **VNT100**  (95% CI) | Sensitivity | Specificity | Sensitivity | Specificity | Sensitivity | Specificity | Sensitivity | Specificity |
|  | 85%  (72.9-92.4) | 98.7%  (92.4-99.9) | 98.1%  (88.4-99.9) | 98.8%  (93.1-99.9) | 85%  (78.3-86.6) | 98.07%  (93.9-99.9) | 97.9%  (90.1-99.9) | 94.7%  (90.7-95.7) |
