# Supplementary material for: Combination of ELISA screening and seroneutralisation tests to expedite Zika virus seroprevalence studies
Source: Virol J. 2018 Dec 27;15:192. doi: 10.1186/s12985-018-1105-5 (PMC6307276; doi:10.1186/s12985-018-1105-5)
Supplement: Supplementary file 6 — Comparison of CPE-based Virus Neutralization Titre100 and PRNT50 titres. (DOCX 172 kb) [file 12985_2018_1105_MOESM6_ESM.docx]

**
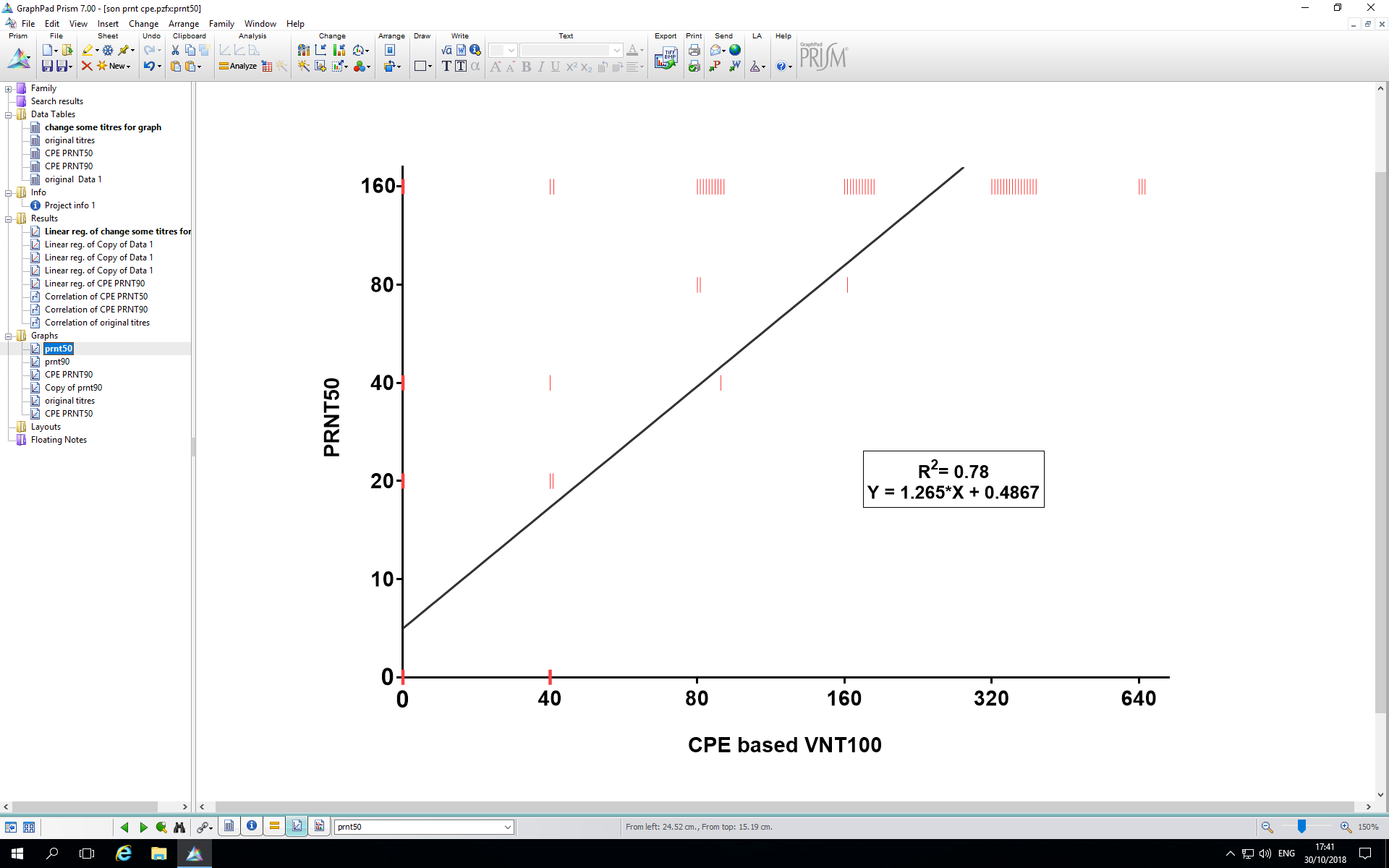
**

**Additional file 6.** Comparison of CPE-based Virus Neutralization Titre100 and PRNT50 titres. Two-tailed p was calculated as 0.003 between PRNT50 and CPE-based VNT100
