# Supplementary material for: Combination of ELISA screening and seroneutralisation tests to expedite Zika virus seroprevalence studies
Source: Virol J. 2018 Dec 27;15:192. doi: 10.1186/s12985-018-1105-5 (PMC6307276; doi:10.1186/s12985-018-1105-5)
Supplement: Supplementary file 7 — Comparison of CPE-based Virus Neutralization Titre100 and PRNT90 titres. (DOCX 168 kb) [file 12985_2018_1105_MOESM7_ESM.docx]

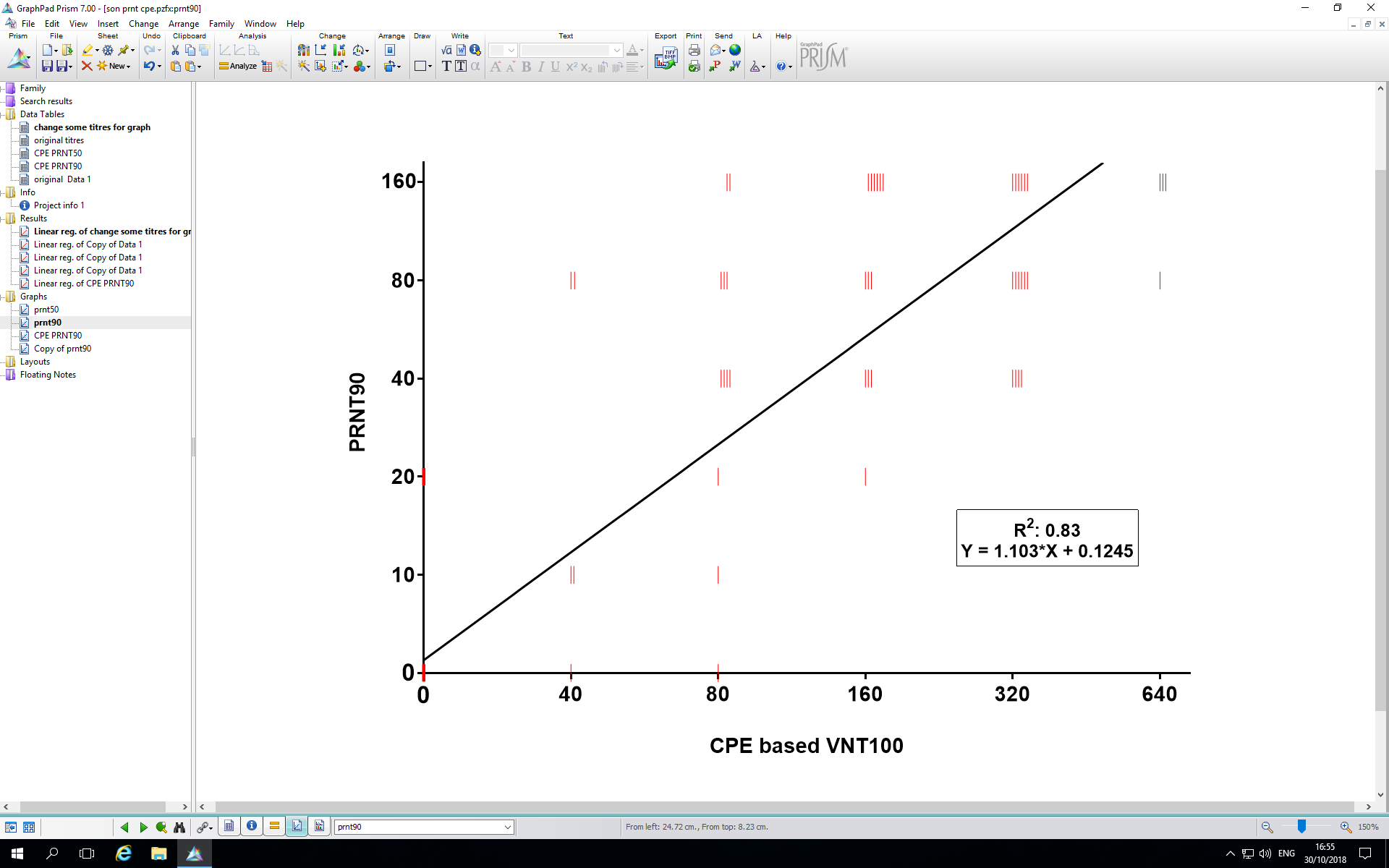


**Additional file 7.** Comparison of CPE-based Virus Neutralization Titre100 and PRNT90 titres. Two-tailed p value was calculated as 0.001 between PRNT90 and CPE-based VNT100
