# Supplementary material for: Combination of ELISA screening and seroneutralisation tests to expedite Zika virus seroprevalence studies
Source: Virol J. 2018 Dec 27;15:192. doi: 10.1186/s12985-018-1105-5 (PMC6307276; doi:10.1186/s12985-018-1105-5)
Supplement: Supplementary file 8 — Specificity and sensitivity of VNT in DENGUE ELISA negative and positive samples. (DOCX 14 kb) [file 12985_2018_1105_MOESM8_ESM.docx]

**Additional file 8**. Specificity and sensitivity of VNT in DENGUE ELISA negative and positive samples

|  | **DENGUE ELISA Negative** | | | **DENGUE ELISA Positive** | |
| --- | --- | --- | --- | --- | --- |
|  | **PRNT90** | | | | |
| **VNT** | Positive (titre≥10) | Negative (titre<10) | Positive (titre≥10) | | Negative (titre<10) |
| Positive (titre≥40) | 2 | 0 | 49 | | 1 |
| Negative (titre<40) | 0 | 47 | 1 | | 42 |
| Sensitivity of VNT (95% CI) | 100 % (2/2) (22.7-100) | | 98% (49/50) (91.6-99.8) | | |
| Specificity of VNT (95% CI) | 100% (47/47) (96.7-100) | | 97.7% (42/43) (90.2-99.8) | | |
