# Supplementary material for: Combination of ELISA screening and seroneutralisation tests to expedite Zika virus seroprevalence studies
Source: Virol J. 2018 Dec 27;15:192. doi: 10.1186/s12985-018-1105-5 (PMC6307276; doi:10.1186/s12985-018-1105-5)
Supplement: Supplementary file 9 — Specificity and sensitivity of VNT in low ZIKV seropositivity (titre 40–80) and strong ZIKV positivity (titre ≥160) compared to the PRNT90. (DOCX 14 kb) [file 12985_2018_1105_MOESM9_ESM.docx]

**Additional file 9**. Specificity and sensitivity of VNT in low ZIKV seropositivity (titre 40-80) and strong ZIKV positivity (titre≥160) compared to PRNT90

|  | **PRNT90** | | |
| --- | --- | --- | --- |
|  | Positive (titre≥10) | | Negative  (titre<10) |
| VNT100 (titre 40-80) | 17 | | 1 |
| VNT100 (negative) | 1 | | 87 |
| Sensitivity of VNT (95% CI) | 94.4 % (17/18) (77.3-99.5 ) | | |
| Specificity of VNT (95% CI) | 98.9% (87/88) (95.4-99.9) | | |
| VNT100 (titre≥160) | 34 | 0 | |
| VNT100 (negative) | 1 | 87 | |
| Sensitivity of VNT (95% CI) | 97.1 % (34/35) (88.7-97.1) | | |
| Specificity of VNT (95% CI) | 100% (87/87) (96.6-100) | | |
